# Extended magenta aurora as revealed by citizen science

**DOI:** 10.1038/s41598-024-75184-9

**Published:** 2024-10-28

**Authors:** Ryuho Kataoka, Sachin Alexander Reddy, Shinya Nakano, Joshua Pettit, Yuki Nakamura

**Affiliations:** 1 National Institute of Polar Research, Tachikawa, Tokyo 190-8518 Japan; 2 SOKENDAI, Hayama, Kanagawa 240-0193 Japan; 3 Graduate School of Science, The University of Tokyo, Hongo, Tokyo 113-0033 Japan; 4 Institute of Statistical Mathematics, Tachikawa, Tokyo 190-8562 Japan; 5 George Mason University, 4400 University Dr, Fairfax, VA 22030 USA; 6 NASA GSFC, Greenbelt, MD 20771 USA

**Keywords:** Aurora, Magnetospheric physics

## Abstract

During large geomagnetic storms, red auroras are typically observed from low-latitude countries such as Japan. The color arises from a specific emission line of oxygen atoms at high altitudes. However, during the May 10-11th 2024 superstorm, magenta auroras were observed above Japan instead of the typical red. In this study, we demonstrate that the magenta hue is created by a mixture of red (O) and a blue (N_2_^+^) aurora at extremely high altitudes. The blue color originates from the N_2_^+^ first negative emission band caused by both resonant scattering of the upwelling molecular ions and heavy particle precipitation during the storm. This study is primarily driven by observations from citizen scientists, and confirmed and quantified with observations from spacecraft and modeling techniques. Additionally, we show that high solar activity, terrestrial season, and the preheating of the atmosphere all contribute to the occurrence of magenta aurora. This study showcases the value and richness of citizen science, and we anticipate that such approaches will become increasingly important in the future.

**Supplementary Information:**

The online version contains supplementary material available at 10.1038/s41598-024-75184-9.

## Introduction

**Fig. 1:**
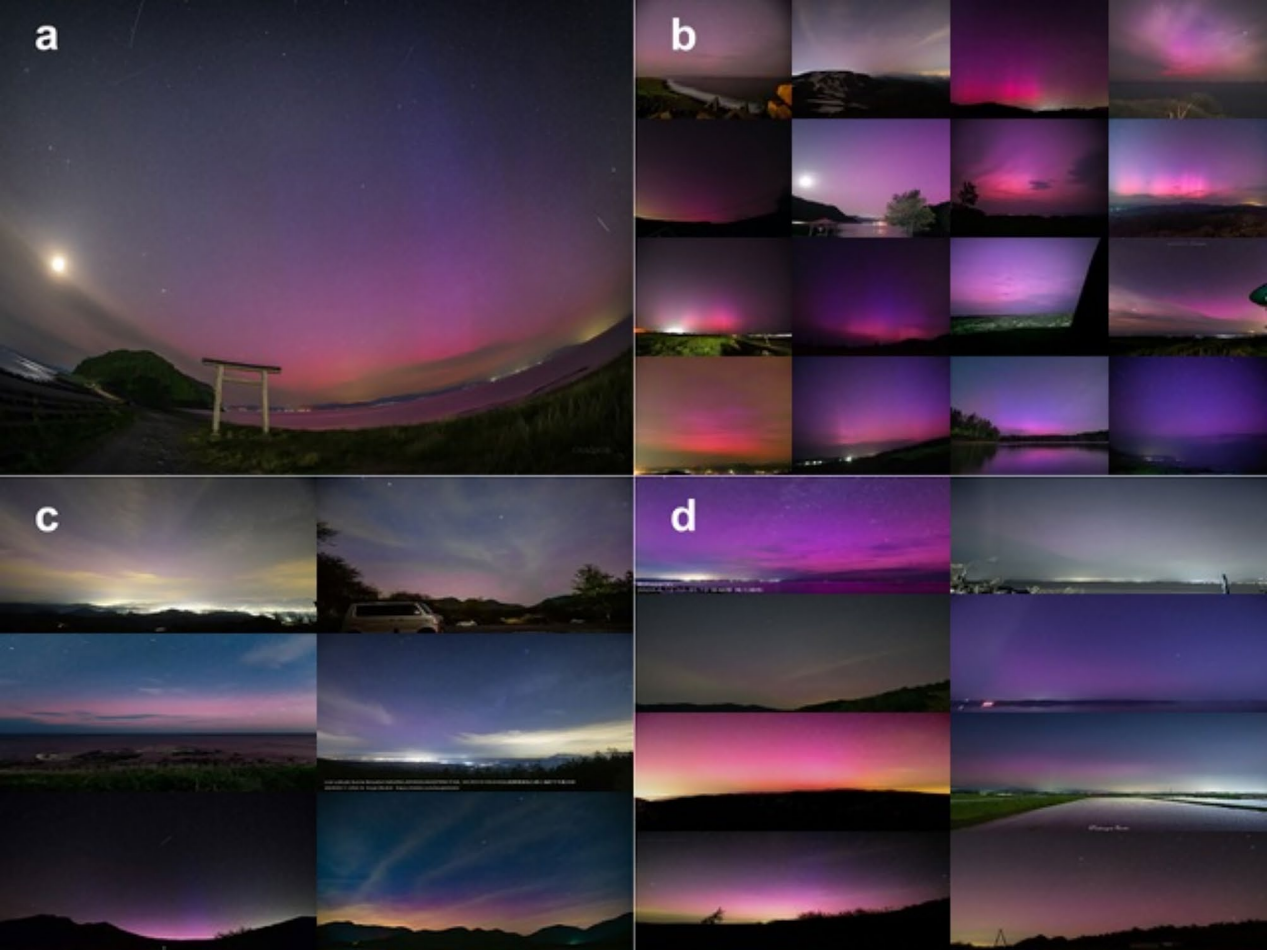
A collage of aurora observed by citizen scientists above Japan between 6 pm JST on 11^th^ May 11 and 6 am JST on 12^th^ May, 2024. **a**, Magenta aurora observed by KAGAYA from Aomori prefecture at 41.00° N and 140.87° E on 11^th^ May, 0934 UT (20:34 JST). Three color structure is clear here; red bottom, purple body, and top blue pillows. **b**, Observed over Hokkaido; GLAT > 42° N. **c**, Observed over Chubu / main island west; GLAT: 35° ~ 38° N. **d**, Observed over Tohoku / main island east; GLAT: 38° ~ 42° N. Note that the magenta hue was robustly observed regardless of individual camera settings or personal image processing. This plot was generated using the Python Imaging Library (PIL) and Pillow package v10.2.0 (https://pillow.readthedocs.io/en/stable/).

In early May 2024, a series of solar flares and subsequent coronal mass ejections (CME) triggered an extreme geomagnetic storm. The 10-11th May 2024 storm ranks 9th in the 110-year history of the Kakioka Magnetic Observatory and 6th in the 66-year history of the Dst index. This super storm caused aurora to appear above low latitudes countries such as Japan. Many people worldwide took great interest in the storm, providing real-time auroral observations on social media platforms such as X. In Japan alone, over a thousand people attempted to view the aurora, leading to one of the densest observation efforts in the world. The public used commercially available cameras to capture the faint auroras that were mostly invisible to the naked eye. Remarkably, the photographs reveal a clear and dominant magenta hue instead of the traditional red typically observed above Japan^3,4^ (Fig. 1). This study aims to investigate the cause of this magenta aurora through photographs from the public, spacecraft observations, and physics-based and statistical modeling.

Utilizing citizen scientists to observe the aurora is a powerful and relatively recent advance in our field^8,9^. Such an approach was used to quantify the unusually bright auroras observed during the December 1st 2023 storm^10^, which was confined to the northernmost parts of Japan, with an order of 10’s of citizen observations. During the May 10-11th 2024 storm, 100’s of citizen observations were made across the full length of Japan (**Method**). To the best of our knowledge, this is the largest number of auroral sightings above Japan during a single event.

The background and the time sequence of events is as follows: from May 7th an unprecedented 5, and possibly more, halo CMEs left the Sun and headed towards Earth. After reaching Earth on May 10th, an extreme geomagnetic storm was triggered. The storm peaked at 0200 UT (1100 Japan Standard Time) on May 11th with the real-time Dst index of -412 nT. This was the largest storm since the 2003 Halloween storms. When combined with a favorable local time and slow recovery phase; this provided Japan with the right conditions to observe the aurorae^11^. The solar wind parameters, storm evolution, and citizen science observations are illustrated in **Extended Data Figure 1**.

**Fig. 2:**
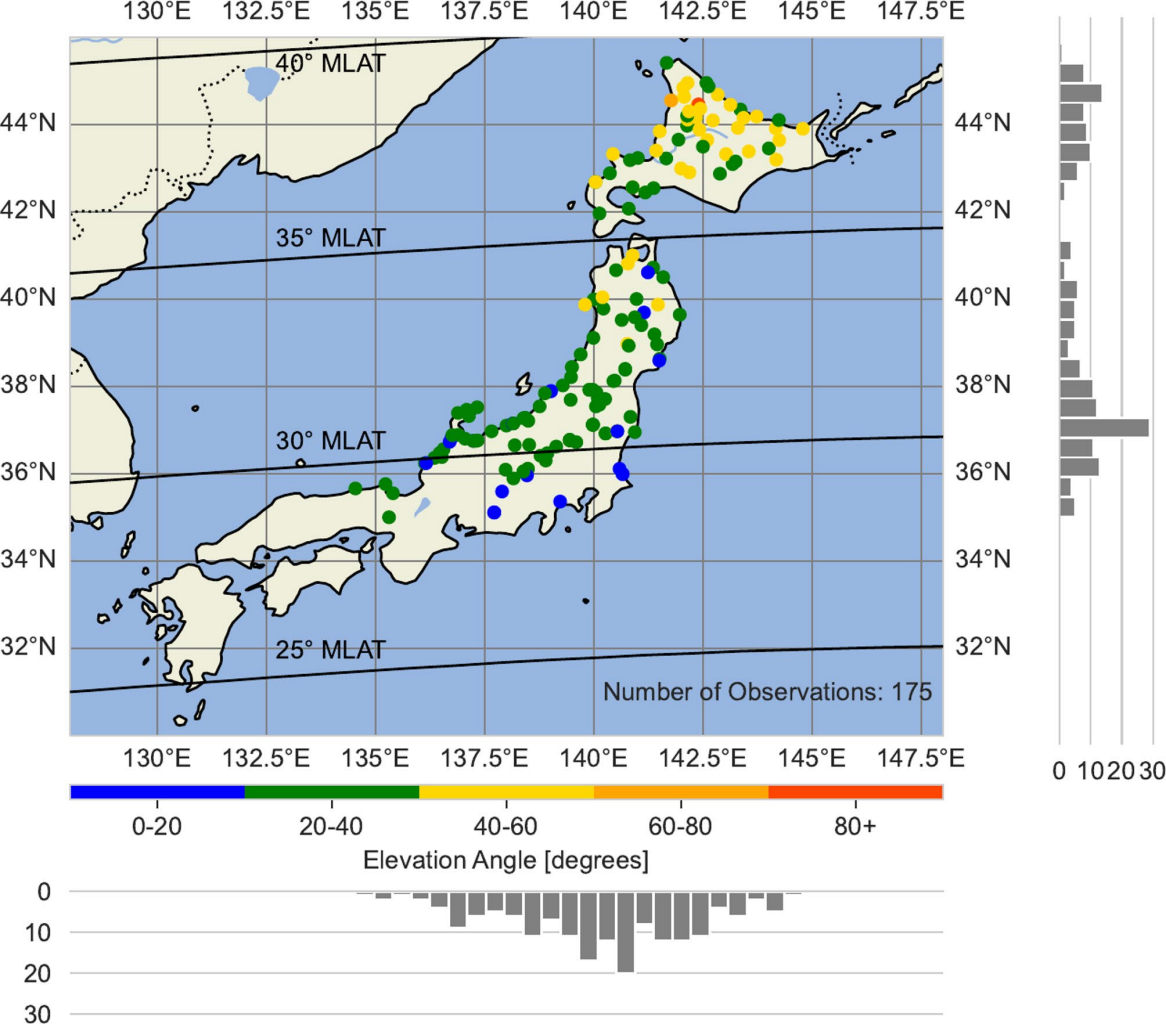
The distribution of 179 auroral observations over Japan during the May 11, 2024 super storm as a function of geolocation and elevation angle. This plot and map was generated with Natural Earth and Cartopy v0.23 (https://scitools.org.uk/cartopy/docs/latest/).

In Fig. 2 we show the distribution of successful citizen science observations of aurora over Japan with respect to elevation angle. As expected, the elevation angles increase with increasing latitude, and we can use these angles to estimate the height of the aurora. All citizen observations were made between 27° N and 46° N geographic latitude (GLAT). **Extended Data Figure 2** shows the total number of observation attempts (775), with “Yes” indicating success and “No” meaning unsuccessful. The time variation of the citizen observations is shown in **Extended Data Figure 3**.

**Fig. 3:**
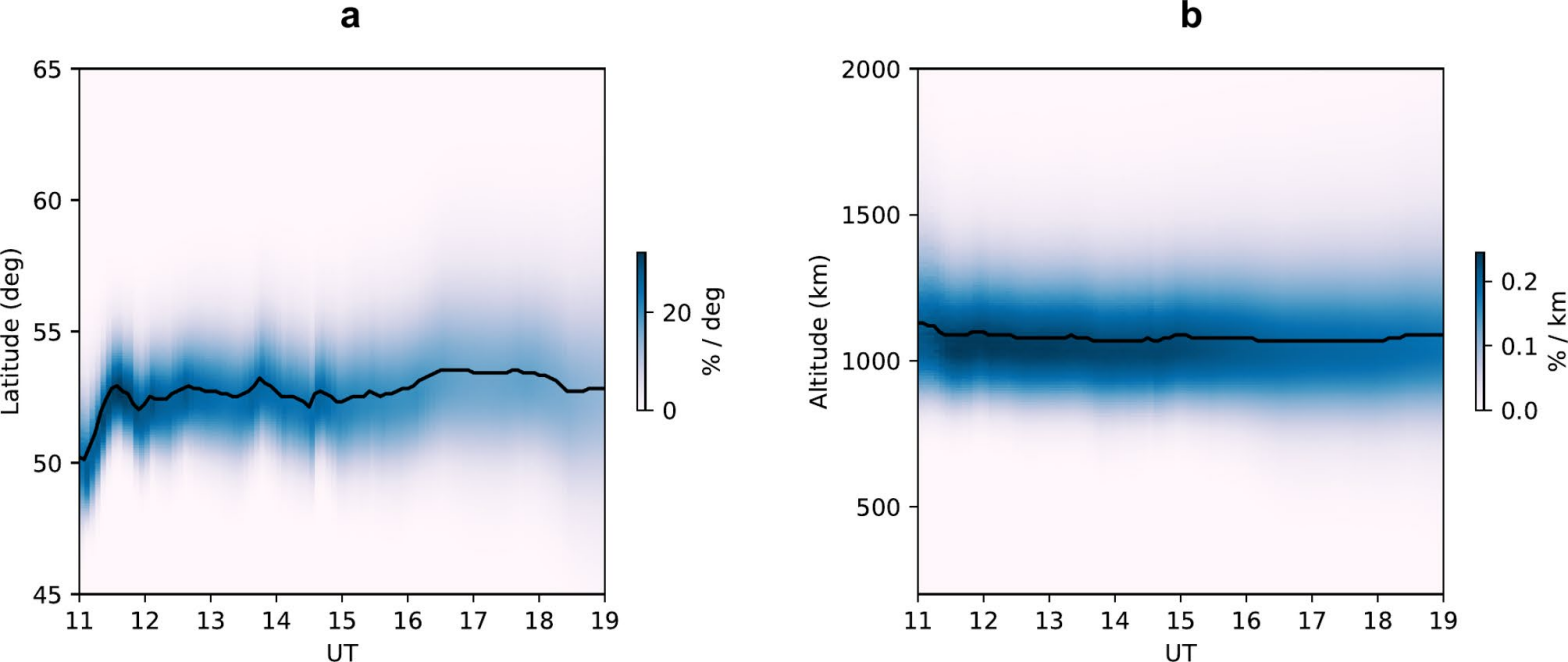
Latitude and altitude estimation using particle Markov chain Monte Carlo method. **a,** Estimated geographic latitude from the elevation angle data points of citizen scientists. **b**, Estimated emission altitude.

In Fig. 3, we use these elevation angles to calculate the time-varying auroral positions using the Particle Markov Chain Monte Carlo method^12^ (**Method**). This analysis revealed a surprisingly high estimate of auroral altitude ~ 1000 km. Starting from the point of 50° ~ 53° N and 140° E at 1000 km, the footprint of magnetic latitude at 100 km is 47° ~ 50° magnetic latitude (MLAT).

The 47 ^o^ ~ 50° MLAT footprint is consistent with the low-energy electron boundary as identified from satellite observations (**Extended Data Figure 4**). The low-latitude extension of the auroral electron precipitation boundary was identified at 45° ~ 48° MLAT before 1130 UT, and at 47° ~ 52° MLAT after 1130 UT around the Japanese meridian. MetOp3, MetOp1, and NOAA18 satellites passed the electron boundaries at 1131UT, 1219 UT, 1305 UT, respectively. There is a small variation of auroral equatorial boundary latitudes after 1130 UT, which is consistent with the reconstructed auroral latitude in Fig. 3.

Now we discuss the cause of the magenta color, given their unusually high altitude. Our hypothesis is that the magenta color is created by the mixture of red and blue aurora as observed from the ground. It has been known since the 1920’s that the sunlit aurora goes up to 1000 km, but that it rarely exceeds this altitude^2^. In May, the northern hemisphere is summer, and this is usually regarded as ‘out of season’ for viewing aurora owing to the bright arctic night. In the middle latitudes there are further contributions from the night sky of the troposphere and the sunlit upper atmosphere. At a geographic position of 52° N and 140° E on the 11th May, the atmosphere above 350 km is sunlit throughout the nightside. Therefore, it can be interpreted that the blue aurora is driven by the resonant scattering of upwelling N_2_^+^ at high altitudes^5^. This is supported by some of the citizen photos which show dazzling blue rays rising from the magenta body.

**Fig. 4:**
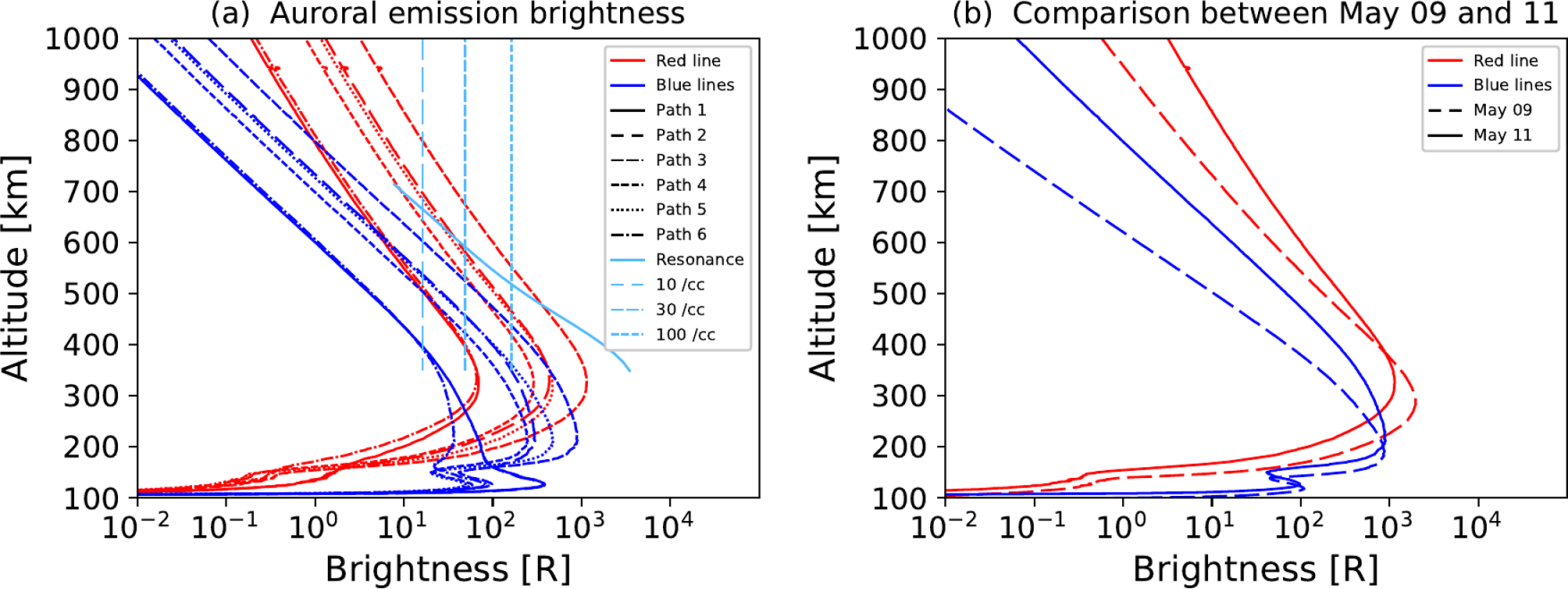
PTRIP simulation results of red and blue auroras. **a**, The red line (630.0 nm) and blue lines (391.4 nm and 427.8 nm) auroral emission profiles with the background atmosphere calculated by the DTM for the condition on May 11^th^ and with electron and H-ENA fluxes obtained by the TED and MEPED instruments, respectively. The solid blue lines show the intensities calculated for electron precipitation only. Paths 1–6 correspond to the passage of MetOp-1 at 10:37 UT, NOAA-18 at 11:22 UT, MetOp-3 at 11:31 UT, MetOp-1 at 12:19 UT, NOAA-18 at 13:05 UT, and MetOp-3 at 13:12 UT. The sky blue denotes the resonance scattering intensity of N_2_^+^ first negative bands. The sky blue curve is the N_2_^+^ resonant scattering intensity as calculated from the GITM’s active-time simulation result, while the dotted lines are the N_2_^+^ resonant scattering intensities for the fixed N_2_^+^ ion densities of 10, 30, and 100 cm^-3^. **b**, Comparison of auroral emission profiles between the two background atmospheres on May 9th and 11^th^ with the same incident electron and H-ENA fluxes during Path 3.

Note here that in the N_2_^+^ 1st negative band, the brightest 391.4 nm emission can be cut-off or significantly reduced in the photographs taken by commercial cameras^13^ which were often utilized by citizens. Our estimation of the blue emission intensity as shown below are the summation of 391.4 nm and 427.8 nm, which can give an overestimation of the resonant scattering. Considering such a significant ambiguity of the citizen science, our estimate of the N_2_^+^ density for this event is on the order of 30 cm^-3^ above 350 km altitude (Fig. 4). The estimated values can compensate for the extremely high-altitude range beyond 600 km, where we cannot refer to the typical N_2_^+^ density as obtained from the GITM simulation result of active time (**Method**). The enhanced N_2_^+^ density at the extremely high altitude, which cannot be captured by GITM, is possibly due to the upwelling N_2_^+^ from the F-region associated with the auroral electron precipitation during magnetically active periods^14,15^, which can also explain the localized blue pillow structure. Note also that our estimated values are well below the historically largest event^16^ of 1000 cm^-3^, and our estimation should be tested and examined in future by satellite observation and modeling works.

Another contributor to the blue emission band is the heavy particle aurora caused by the atmospheric precipitation of the energetic ions and neutrals^6,7^. Here, ring current ions of > tens of keV charge-exchange with the geocorona, enabling the storm recovery phase as well as driving precipitation into the atmosphere. Figure 4 shows the results of our PTRIP (Particle TRansport In Planetary atmospheres) simulation^17^ (**Method**). The N_2_^+^ emission from the heavy particles can only contribute at relatively low altitudes, such as in the E region. At altitudes of > 200 km, the heavy-particles that drive the blue hue, contribute less than the low-energy (< 1 keV) precipitating electrons (**Extended Data Figure 5**). Note that the atmospheric extinction is most effective for blue emissions especially at low elevation angle, i.e. at low altitude in this in this case of geometry. This means that the emission intensities of blue colors can be stronger than observed especially at low altitude, which is consistent with our simulation results. Therefore, we can conclude that the relative contributions of the particle impact against to the resonant scattering is significantly small for high altitudes.

Extremely high-altitude (~ 1000 km) red aurora is more surprising, because they typically only extend up to 600 km and are generally driven by broadband electrons^3^. To extend the subvisual aurora by an order of 10 R up to 1000 km, an extreme and rare expansion of the atmosphere is required. This preheating of the middle atmosphere is possible because it is roughly 10 h after the storm peak as confirmed by our CCMC DTM runs (**Method**). Figure 4 shows our PTRIP simulation results, which reproduces > 10 R red auroras up to 800 km. For > 10 R red aurora to extend up to 1000 km, the upper atmosphere needs to be denser and hotter. A stable auroral red arc^4^ is another possible contributor to the intensity of high-altitude red, although this alone cannot explain the ray-like structures that appear in many of the photos. The possible role of SAR arc is discussed for the main phase of the same storm based on citizen science^18^. As a different mechanism, for example, the storm-time substorm is suggested as the cause of the red ray-like structure as observed from the low-latitude area^10^. We therefore conclude that preheating of the middle latitude atmosphere, was likely another important contributor to the magenta body, in addition to the season and high solar activity.

Finally, it is important to note that the subvisual intensity of the magenta aurora was a key factor in our ability to clearly identify and elucidate this phenomenon in modern times. In Japan’s historical records, which span over a millennium, auroras observed with the naked eye were described as a “red sign”^1^. The most extreme auroral event in Japan occurred in September 1770 and was visible from all regions of Japan, even appearing above Kyoto, which was at a magnetic latitude of 24° at that time^19^. It is estimated that the magnitude of the September 1770 storm was comparable to, or exceeded that of, the historically significant Carrington Event of 1859. In comparison, the amplitude of the storm of May 2024 was relatively weak, with auroras only faintly visible to the naked eye from the northern parts of Japan. Interestingly, the preheating of the atmosphere reduces the peak brightness of the aurora, as shown in Fig. 4

We conclude, by reiterating the importance of using citizen scientists in auroral investigations. A great majority of people now own camera-phones that have a greater sensitivity than the naked eye and they share their images in real-time via social media. Paired with the continuous advance of AI in automatically interpreting questionnaires, identifying color in images, and translating languages, we expect the role of the public to only increase in any scientific investigations. We would also like to emphasize that this study was primarily conducted in Japanese and that language across citizen activities is less of a barrier to novel science.

## Methods

### Data collection via SNS, cleaning, and elevation angle analysis

To collect the citizen science data, Ryuho Kataoka first asked the X (formerly Twitter) community to attempt to observe the aurora and to tag it with a specially created hashtag, which means ‘aurora-citizen’ in Japanese. The second phase involved asking the X community to fill out a questionnaire which contained the following fields: 1) email; 2) observation location (city, latitude, longitude); 3) observation time; 4) elevation angle; 5) evidence of photo, (generally a link to your X account); 6) your name; 7) any additional comments. There was a total of 775 submissions to the questionnaire. Elevation angles were calculated by cross checking the auroral height with star constellations (e.g. Polaris, Cassiopeia, etc.). We also received submissions from outside of Japan, but they were removed from the dataset. We hope to expand such a study to a global scale in future works.

### Particle Markov Chain Monte Carlo method

To estimate the height of the aurora we performed a Bayesian analysis with the Particle Markov chain Monte Carlo (PMCMC) method^12^. First, we consider the following state transition model:

1 $$\lambda_{k} = \lambda_{k - 1} + v_{\lambda ,k} ,$$

2 $$a_{k} = a_{k - 1} + v_{a,k} ,$$

where *λ*_*k*_ and *a*_*k*_ denote the latitude and altitude of the emission source at a time step *k*, respectively.

The variations of *λ*_*k*_ and *a*_*k*_ are represented by* v*_*λ,k*_ and *v*_*a,k*_, respectively. Assuming that the aurora is uniform in longitude and that the Earth is a true sphere, the elevation angle *y*_*ki*_ observed at a latitude *Λ*_*i*_ is obtained as

3 $$y_{ki} = \arctan \left( {\frac{{\cos \left( {\lambda_{k} - \Lambda_{i} } \right) - R_{{\text{E}}} /\left( {R_{{\text{E}}} + a_{k} } \right)}}{{\sin \left( {\lambda_{k} - \Lambda_{i} } \right)}}} \right) + \varepsilon_{ki} ,$$

where *ε*_*ki*_ is an observation noise. We calculate the posterior distibution of *λ*_*k*_ and *a*_*k*_ given the elevation angle data with the PMCMC method^10^ in which we estimate the hyperparameters with the Metropolis–Hastings method^20^. *v*_*λ,k*_, *v*_*a,k*_, and *ε*_*ki*_ are assumed to obey independent normal distributions whose mean is zero. The variances of the three variables were treated as hyperparameters in the analysis.

### Resonance scattering of N_2_^+^ ions

To estimate the resonance scattering intensity of N_2_^+^ first negative bands, we used the g-values of 0.041 and 0.013 photons sec^-1^ ions^-1^ for 391.4 nm and 427.8 nm, which is defined as the number of photons that are resonance scattered by an N_2_^+^ ion per second when illuminated by unattenuated sunlight^21,22^. We also assumed a column integration width of 300 km. Although the simulation run of Global Ionosphere Thermosphere Model (GITM) for the May 11 storm has not been ready in CCMC, we consulted the altitude profile of the N_2_^+^ ion density from the accumulated data of CCMC/GITM runs in the recovery phase of the June 23 storm, which occurred during a similar UT, season, and solar activity, at 140°E and 55°N. The GITM is a 3D physics-based model that explicitly solves for 7 neutral and 7 ion species. GITM is on an altitude grid (instead of a pressure grid like most thermosphere/ionosphere models), that extends from 90 km through 600 km. We utilized version 21.11 with resolution of 2° latitude and 4° longitude.

### PTRIP modifications

PTRIP is a Monte Carlo model that solves the transport of electrons, protons, and hydrogen atoms in planetary atmospheres to calculate auroral emission profiles^17^. To apply PTRIP to simulate Earth’s auroral emissions, we have implemented the collisional cross sections for the O(^1^S) and O(^1^D) excitation due to electron impact on atomic oxygen^23^, vibrational excitation cross sections of molecular nitrogen by electron impact^24^, and differential elastic cross sections of molecular nitrogen for proton impact based on laboratory measurements^25^. The volume emission rate of N_2_^+^ first negative bands at 391.4 nm due to electron impact is estimated by the emission cross section^24^, and the proton and H-ENA impacts are estimated by multiplying the production rate of N_2_^+^ by a factor of 0.07 based on laboratory measurements for the electron-impact emission cross section^26,27^. The volume emission rate of N_2_^+^ first negative bands at 427.8 nm is estimated by multiplying the volume emission rate of 391.4 nm by a factor of 0.35, again based on laboratory measurements^28^. We also consider the quenching effect for the 630.0 nm emission from the atomic oxygen due to the collision with N_2_, O_2_, and O^29,30^. We assumed the layer thickness of 300 km (~ 3 deg, as suggested from satellite observations) and the observation perpendicular to the emission layer, resembling the observation geometry.

Model inputs to the PTRIP are atmospheric density profiles and incident fluxes of precipitating particles. MEPED 0 deg ions and TED 0 deg electrons are used for the incident fluxes of H-ENAs and electrons, respectively. The altitude profiles of N_2_, O_2_, and O used for the background atmosphere are obtained from the Drag Temperature Model (DTM). The DTM is a semi-empirical model that utilizes the F10.7 index and the Kp index to calculate the neutral temperatures and densities from the surface to 1000 km. It takes advantage of the CHAMP and GRACE missions to constrain the model-inferred densities. The version used in this study is DTM 2020.

## Electronic supplementary material

Below is the link to the electronic supplementary material.

Supplementary Material 1

## Data Availability

POES/MetOp satellite are available from: https://www.ngdc.noaa.gov/stp/satellite/poes/dataaccess.html. Simulation runs are available from CCMC: https://ccmc.gsfc.nasa.gov/results/viewrun.php?domain=IT&runnumber=Josh_Pettit_052224_IT_1. https://ccmc.gsfc.nasa.gov/results/viewrun.php?domain=IT&runnumber=Josh_Pettit_052224_IT_2. https://ccmc.gsfc.nasa.gov/results/viewrun.php?runnumber=Amol_Kishore_071623_IT_4.
