# Supplementary material for: Extended magenta aurora as revealed by citizen science
Source: Sci Rep. 2024 Oct 28;14:25849. doi: 10.1038/s41598-024-75184-9 (PMC11519668; doi:10.1038/s41598-024-75184-9)
Supplement: Supplementary file 1 — Supplementary Material 1 [file 41598_2024_75184_MOESM1_ESM.pdf]

Extended Data Figure 1

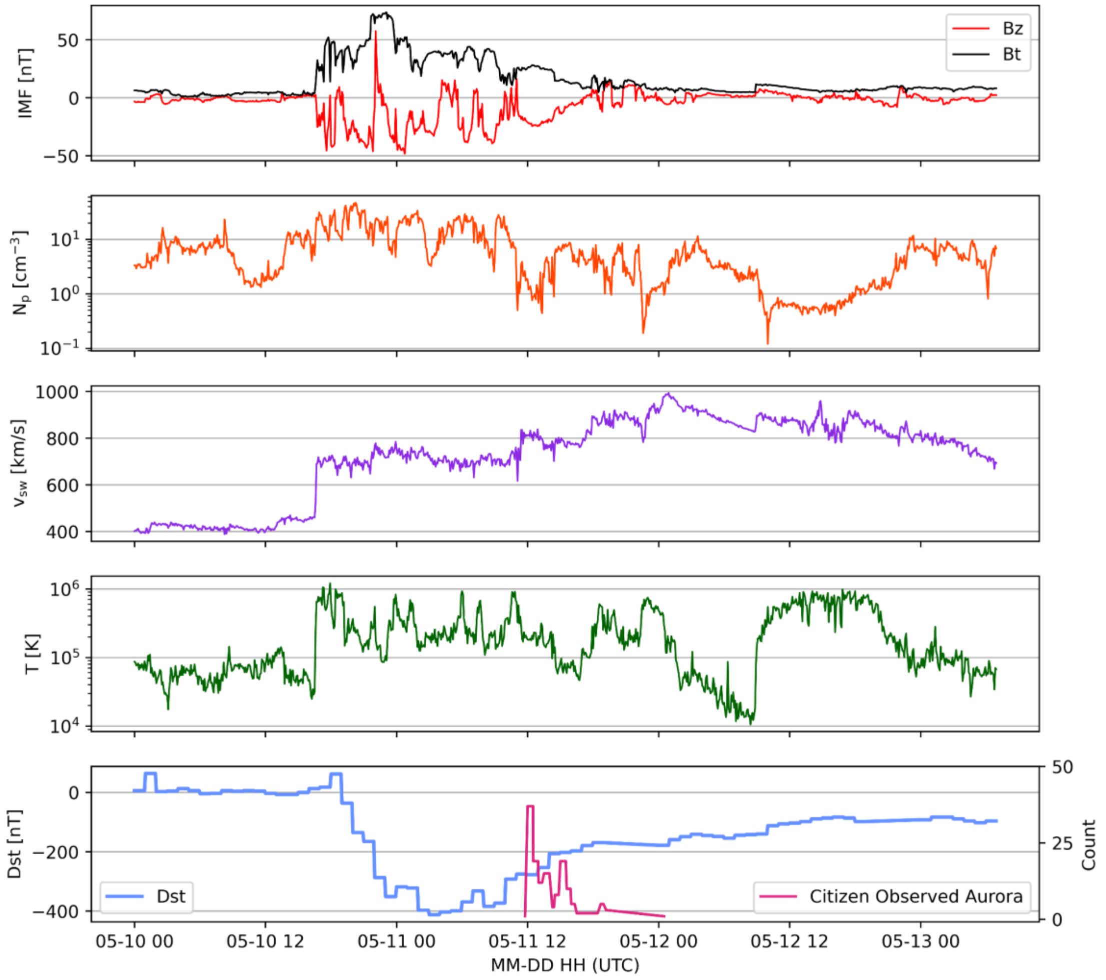

Extended Data Figure 1. The solar wind parameters (interplanetary magnetic field, proton number density, solar wind speed, and proton temperature) and the real-time Dst index with the number of citizen scientist observations. The interplanetary shock arrived at 1630 UT on May 10, 2024, and the storm peaked at 0200-0300 UT on May 11, 2024. The auroral observation in Japan begin around 1200 UT on May 11, 2024 during the early recovery phase of this super storm.

Extended Data Figure 2

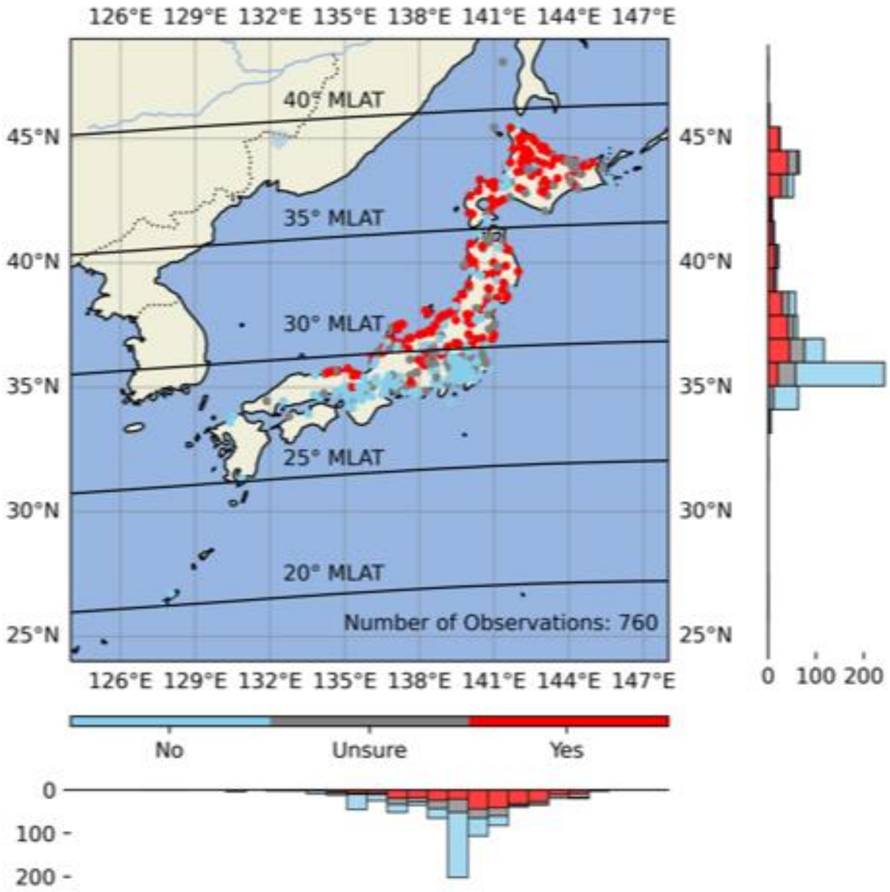

Extended Data Figure 2. All citizen science data. A large cluster is observed around 37° N, with limited observations between 38° N and 42° N. This is somewhat in line with the population of people in Japan. This plot and map was generated with Natural Earth and Cartopy v0.23.

Extended Data Figure 3

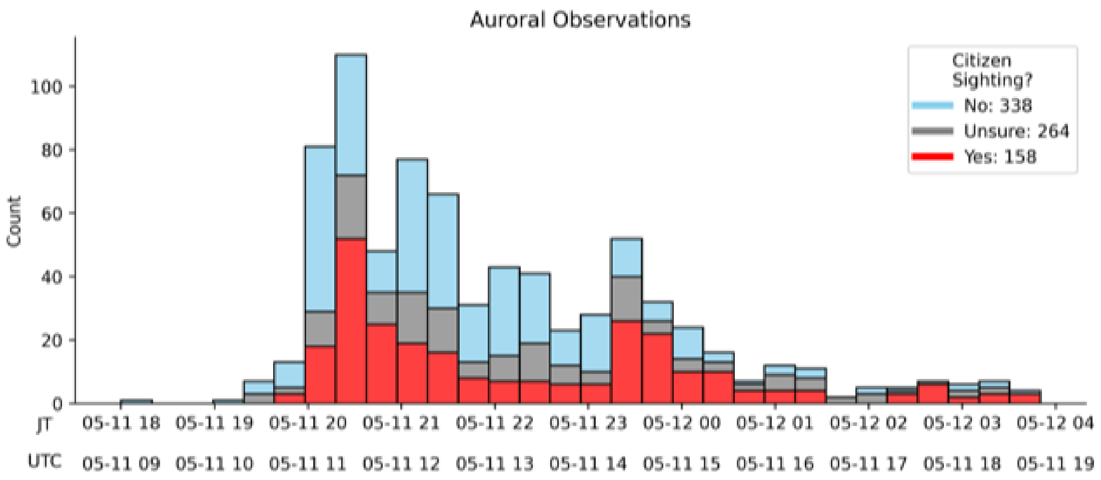

Extended Data Figure 3. Observations of aurora submitted by the general public during the May 2024 super storm. This plot was generated using Seaborn v0.12.2.

Extended Data Figure 4

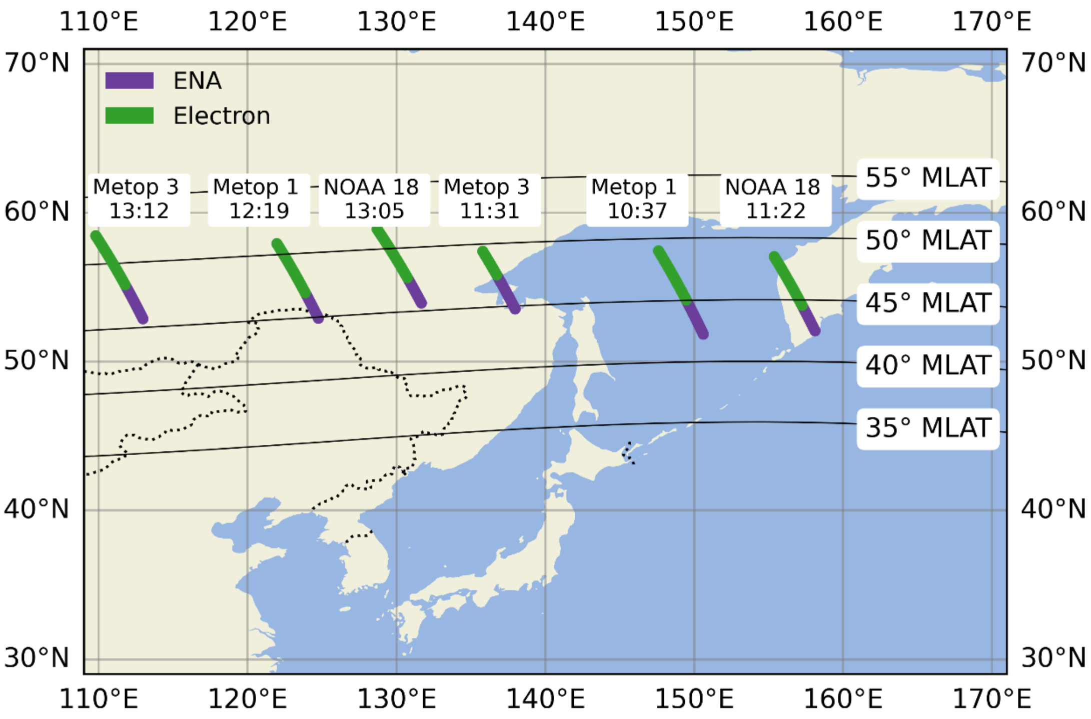

Extended Data Figure 4. MetOp1-3 and NOAA18 orbit tracks with the electron boundaries and ENA boundaries. The TED 0-deg electron fluxes are used to identify the electron boundaries (green) between 10 and 14 UT. The MEPED 27 keV protons are also used to identify the proton/ENA boundaries. The boundaries are not sharp, and have ~300 km width. The jump from NOAA 18 (1122 UT) to MetOp3 (1131 UT) is significant, related to the spatial variation rather than the temporal variation. The general tendency of increasing boundary latitudes are consistent with the storm recovery. This plot and map was generated with Natural Earth and Cartopy v0.23.

Extended Data Figure 5

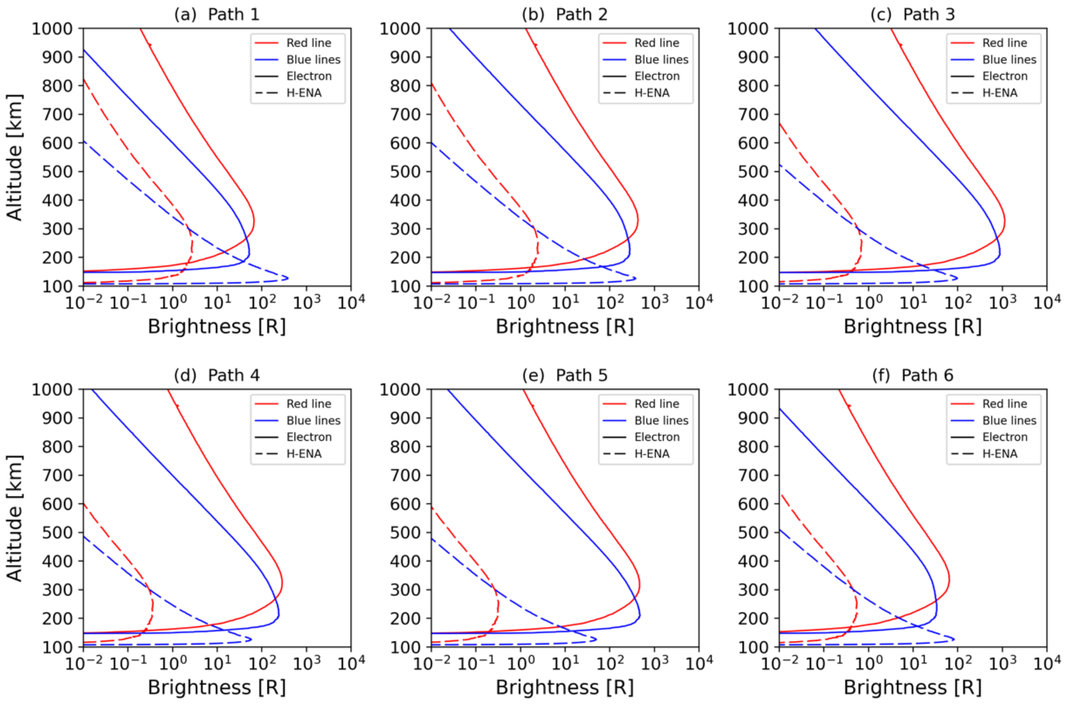

Extended Data Figure 5. The simulated red line (630.0 nm) and blue lines (391.4 nm and 427.8 nm) auroral emission profiles for Paths 1-6. Solid curves represent the emissions due to electron precipitation, and dashed curves are the emissions due to H-ENA precipitation.

Supplemental Data Table 1

Supplemental Data Table 1. The auroral observation list of citizen scientists. Only "successful" observations are listed here to show their measurement of the elevation angle.

UserID	GLAT	GLON	Elevation	Y or N	Time
2	38.639	141.510	30	Y	2:45
9	44.355	143.364	30	Y	21:36
11	38.128	140.449	25	Y	22:00
12	43.906	144.786	43	Y	1:07
16	36.118	138.486	20	Y	1:00
17	38.973	141.445	30	Y	20:22
18	42.914	142.189	42	Y	21:03
25	43.228	141.656	25	Y	20:25
26	42.890	140.370	35	Y	20:28
28	35.997	140.656	18	Y	23:53
30	43.980	142.139	30	Y	20:26
31	40.823	140.784	40	Y	20:26
41	44.687	142.841	59	Y	20:26
47	36.764	137.236	15	Y	23:38
48	44.122	142.154	40	Y	20:30
56	43.000	142.000	55	Y	20:30
63	39.788	140.216	25	Y	21:45
65	37.703	139.468	20	Y	0:22
68	42.690	140.040	35	Y	22:30
74	35.119	137.719	10	Y	23:29
76	36.779	139.451	15	Y	0:09
77	38.600	141.500	13	Y	2:23
95	40.732	141.357	30	Y	21:30
100	43.463	144.010	35	Y	22:30
103	43.406	141.432	40	Y	21:33
105	43.917	144.170	45	Y	20:30
109	43.855	141.510	40	Y	23:32
110	36.251	136.130	22	Y	23:58
112	44.097	142.726	50	Y	20:49

119	35.118	137.719	10	Y	23:50
126	44.170	142.340	20	Y	20:29
130	36.118	138.486	20	Y	0:15
140	36.479	138.937	30	Y	20:40
145	36.930	140.274	20	Y	21:00
147	43.888	142.420	40	Y	20:30
153	43.097	143.184	35	Y	21:00
164	44.961	142.583	30	Y	20:30
165	36.064	138.393	25	Y	23:41
167	40.624	141.237	18	Y	20:30
170	36.365	136.347	20	Y	22:50
171	43.659	141.937	30	Y	20:30
173	44.109	144.246	35	Y	9:30
176	37.129	139.976	20	Y	0:45
179	44.563	141.778	40	Y	20:35
182	36.773	137.343	20	Y	21:50
183	43.202	144.183	55	Y	22:34
187	36.778	139.451	25	Y	2:35
198	36.957	140.944	30	Y	23:40
200	38.979	140.772	45	Y	20:08
206	39.646	141.968	30	Y	0:00
209	37.314	140.838	28	Y	22:24
215	38.742	139.704	35	Y	20:27
227	43.333	143.033	40	Y	20:36
235	37.129	139.976	20	Y	20:45
243	35.118	137.719	15	Y	23:52
244	37.551	138.764	20	Y	1:25
246	36.393	136.525	20	Y	21:54
247	44.639	142.070	40	Y	20:20
254	36.779	139.451	20	Y	3:12
270	36.670	138.518	20	Y	20:07
271	37.724	140.256	20	Y	19:46
274	44.851	142.050	40	Y	20:33
275	37.590	140.125	23	Y	20:55
278	37.115	138.005	20	Y	23:30
280	42.691	140.040	45	Y	20:31

281	37.751	140.092	23	Y	21:47
285	37.872	140.059	20	Y	22:51
286	43.334	140.444	50	Y	21:20
287	35.972	138.472	12	Y	3:20
289	37.933	139.981	26	Y	23:29
290	35.774	135.225	25	Y	23:50
295	37.930	139.902	25	Y	0:17
297	36.930	140.273	25	Y	20:00
299	42.566	140.893	40	Y	20:30
302	37.929	139.903	22	Y	0:36
305	38.410	140.720	20	Y	20:32
306	44.327	142.368	70	Y	20:01
310	42.566	140.893	30	Y	20:30
316	36.249	136.160	10	Y	0:30
319	44.108	144.243	30	Y	20:42
320	36.064	138.393	20	Y	23:36
321	40.000	140.000	20	Y	20:30
324	36.982	140.530	40	Y	20:55
326	35.666	134.538	35	Y	20:12
328	37.529	137.326	36	Y	20:00
331	37.129	139.976	25	Y	20:30
333	36.764	137.236	20	Y	22:40
336	37.550	140.050	20	Y	21:00
339	43.925	143.299	56	Y	20:48
340	36.758	137.297	20	Y	23:30
346	39.526	140.641	30	Y	20:58
347	36.662	138.193	22	Y	21:18
355	35.600	137.900	15	Y	23:00
358	44.192	143.726	45	Y	20:03
361	44.169	142.339	45	Y	23:42
371	43.162	143.247	30	Y	20:31
382	43.194	140.825	25	Y	21:00
386	37.902	139.023	15	Y	22:15
392	43.648	142.597	40	Y	20:30
393	42.879	142.889	39	Y	21:58
395	38.136	140.483	30	Y	22:00

397	36.847	136.752	15	Y	22:38
399	36.980	140.536	20	Y	2:46
400	39.704	141.153	17	Y	20:33
401	42.550	141.367	30	Y	20:22
403	36.480	136.472	20	Y	23:25
407	36.979	140.538	15	Y	2:50
414	44.448	142.372	45	Y	20:28
422	44.229	142.173	55	Y	23:25
424	36.725	139.598	20	Y	20:30
426	42.076	140.801	35	Y	20:04
427	36.840	136.750	14	Y	20:50
428	37.219	138.497	35	Y	23:27
429	38.224	139.481	20	Y	20:34
437	36.124	140.592	10	Y	2:57
441	44.466	142.395	90	Y	20:00
442	43.648	144.247	43	Y	21:30
443	35.372	139.227	15	Y	20:25
453	44.212	142.135	25	Y	23:39
454	35.560	135.398	25	Y	0:51
458	36.778	139.451	20	Y	21:10
459	37.161	138.148	12	Y	23:16
465	40.051	140.197	40	Y	0:30
468	37.129	139.976	35	Y	11:50
472	35.995	140.657	10	Y	23:25
483	37.129	139.976	24	Y	2:48
484	35.006	135.313	20	Y	20:00
485	36.900	136.900	20	Y	21:00
487	36.416	138.777	30	Y	20:00
500	37.162	138.163	20	Y	23:30
518	36.772	139.447	30	Y	21:00
525	44.958	142.150	50	Y	21:30
526	41.967	140.133	35	Y	20:00
529	37.401	136.891	20	Y	21:30
535	38.454	139.496	20	Y	23:32
536	38.944	140.805	20	Y	21:30
552	36.566	136.556	20	Y	0:06

553	38.454	139.496	30	Y	23:37
564	38.394	140.719	30	Y	20:10
570	39.883	139.800	45	Y	20:30
574	44.880	142.630	30	Y	23:32
576	43.500	142.500	30	Y	20:30
582	35.899	138.164	28	Y	23:45
591	38.034	139.294	30	Y	0:00
593	39.411	141.087	20	Y	20:45
595	36.736	136.696	10	Y	23:30
599	36.985	137.659	25	Y	23:22
602	36.812	137.046	30	Y	23:50
603	40.513	141.586	20	Y	2:41
611	36.886	136.764	25	Y	23:30
619	45.425	141.667	30	Y	22:48
620	39.593	140.941	20	Y	20:40
622	36.311	138.896	20	Y	0:30
623	44.465	143.131	50	Y	20:35
629	41.010	140.890	40	Y	22:30
631	39.122	139.990	20	Y	20:29
632	37.902	139.023	15	Y	23:42
639	40.007	140.980	30	Y	20:27
661	38.330	138.487	25	Y	11:35
676	44.306	142.184	55	Y	23:00
688	39.198	141.394	25	Y	23:07
690	43.238	141.009	35	Y	20:30
691	44.159	143.433	50	Y	21:01
700	39.877	141.469	48	Y	20:34
702	36.097	137.981	25	Y	23:35
715	39.200	141.400	40	Y	8:30
716	37.846	138.881	25	Y	20:35
717	37.129	139.976	36.5	Y	23:56
722	44.370	142.444	52	Y	20:53
729	44.564	141.772	75	Y	23:25
732	37.283	138.391	20	Y	23:30
746	37.467	137.087	24	Y	20:40
747	37.335	137.137	31	Y	0:20

748	37.467	137.087	21	Y	23:28
750	36.630	139.140	25	Y	20:58
753	42.448	141.178	35	Y	21:06
756	40.673	140.511	35	Y	21:08
762	37.294	138.427	30	Y	20:17
769	43.393	143.552	50	Y	20:45
